# Influence of Dendritic Cells on Viral Pathogenicity

**DOI:** 10.1371/journal.ppat.1000384

**Published:** 2009-07-31

**Authors:** Giulia Freer, Donatella Matteucci

**Affiliations:** Retrovirus Center and Virology Section, Department of Experimental Pathology, University of Pisa, Pisa, Italy; University of British Columbia, Canada

## Abstract

Although most viral infections cause minor, if any, symptoms, a certain number result in serious illness. Viral disease symptoms result both from direct viral replication within host cells and from indirect immunopathological consequences. Dendritic cells (DCs) are key determinants of viral disease outcome; they activate immune responses during viral infection and direct T cells toward distinct T helper type responses. Certain viruses are able to skew cytokine secretion by DCs inducing and/or downregulating the immune system with the aim of facilitating and prolonging release of progeny. Thus, the interaction of DCs with viruses most often results in the absence of disease or complete recovery when natural functions of DCs prevail, but may lead to chronic illness or death when these functions are outmanoeuvred by viruses in the exploitation of DCs.

## Introduction

**Figure 1:**
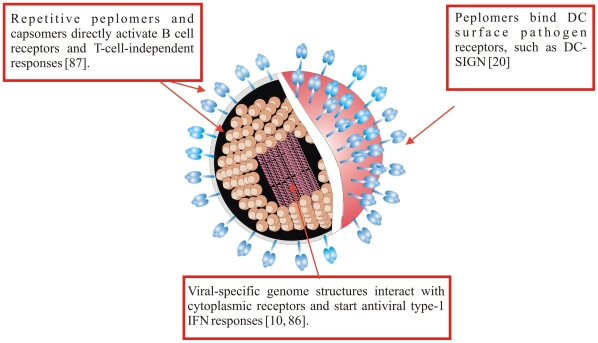
Viral patterns that activate the immune system. A virion can be envisioned as a concentration of different patterns recognized by the immune system: virtually all of its components can be used to start a response before the T cell compartment is active.

Viruses are major targets of the immune system. A variety of viral pathogen-associated molecular patterns (PAMPs), such as the high repetition of capsomers and/or peplomers on virion surface, the production of unique RNA replication intermediates and genome modifications, and others, are recognized as markers of viral invasion by responsive molecules on immune effector cells (see Figure 1). The integration of stimuli delivered by different viral PAMPs leads to inflammation and immune activation which, in turn, are key components of both pathogenesis and recovery from viral infection. Dendritic cells (DCs) possess properties and abilities enabling them to act as unique immune “live adjuvants” [1]. Like no other antigen-presenting cell, they can perform multiple immunogenic tasks, including i) priming of naïve T cells by the expression of special costimulatory surface molecules; ii) cross-presentation, that is, presentation of exogenous antigens in the context of MHC class I molecules to CD8^+^ T lymphocytes, in addition to presentation of MHC class II-restricted peptides; and iii) polarizing naïve T cells to various Th phenotypes. DC activity is normally triggered by pathogens via a variety of receptor molecules and includes the release of distinct interleukins (ILs) directed at regulating T cell differentiation [2]. Indeed, the secretion of soluble mediators seems to be responsible for Th phenotype differentiation and is now considered “signal 3”, after MHC-peptide recognition (signal 1) and contact of costimulatory molecules (signal 2) [3]. The secretion of IL-12, IL-18, tumor necrosis factor (TNF)-α, and interferon (IFN)- α is associated with skewing naïve T cells towards a Th1 phenotype, while Th2 cells are produced in the absence of such cytokines and in the presence of IL-10. In addition, transforming growth factor (TGF)-β induces Foxp3 and promotes the generation of CD25^+^ CD4^+^ T regulatory (Treg) cells, while IL-6 inhibits the generation of Treg cells and induces the production of T helper 17 (Th17) cells, suggesting another reciprocal developmental pathway for CD4^+^ T cells [4]. In turn, Treg cells hinder DCs and/or normal CD4^+^ T cells in their activities, while Th17 cells are involved in inflammatory and autoimmune reactions [5].

Due to different origin, distribution in tissues, and expression of surface receptors, DCs have been divided into conventional (cDCs) and plasmacytoid DCs (pDCs); they differ in immunomodulatory functions and preferentially react to distinct microbial stimuli. cDCs, also known as myeloid DCs, include migratory cells and lymphoid-resident cells that cooperate and are essential to one another to turn on a T cell response. cDCs are mostly devoted to taking up antigen in their steady state and presenting it to T cells in their activated or mature state. Recent evidence shows that early antigen presentation by lymphoid-resident DCs initiates activation and trapping of antigen-specific T lymphocytes in the draining lymph node, while migratory DCs interact with such T cells to induce clonal expansion [6]. In addition, cDCs can recognize cytoplasmic viral RNA by endosomal Toll-like receptor (TLR)-3, binding double-stranded RNA [7], and cytoplasmic receptors such as retinoic-acid-inducible protein I (RIG-I), a helicase that has been shown to bind 5′ triphosphorylated ends in single-stranded RNA present only in certain viral genomes, and melanoma-differentiation-associated gene 5 (MDA5), and start type I IFN production [8]–[10]. To study human DCs, cDCs generated in vitro by differentiation of peripheral blood monocytes in the presence of specific cytokines have been extensively used in many studies. However, the results obtained with in vitro-generated cDCs must be interpreted with caution since recent evidence indicates that in vitro conditions may select at least one specific subset [11]. In this respect, there will probably be abundant new insight into the field brought by studies on humanized mice that exhibit long-term systemic human T cell reconstitution in vivo, allowing for the manifestation of the differential response by human DCs to different pathogens [12].

DCs of the second subset, pDCs, seem to be designed to be part of the first line of defense against viral and intracellular parasite replication: they express endosomal nucleic-acid sensing TLR-7, TLR-8, and TLR-9, that, once triggered, are mostly responsible for the release of type I IFN by these cells [13]. Very recently, however, pDCs were shown to also be capable of cross-presenting viral antigens and to behave as “ready-made stores of MHC class I” that can be charged in endosomes independently of proteasome activity; pDCs are therefore also able to quickly prime CD8^+^ cytotoxic activity upon interaction with virus [14]. pDCs can be normally found in blood and lymphoid organs and can be purified in small numbers from human peripheral blood where they are recognized as CD123^+^, CD11c^−^ cells. As will be discussed further on, most of the data on their role in vivo indicate that a reduction in pDC response is inversely correlated with severity in viral diseases.

To present antigen to T cells, DCs must move from their locations to lymph nodes. The ability to migrate to lymph nodes, as well as to express costimulatory molecules responsible for activating naïve T cells in germinal centers, is acquired through maturation. Such a state, also called activation, is achieved after virus uptake by triggering one of two kinds of receptors: i) receptors recognizing PAMPs, including TLRs on the cell surface and endosomes, as well as cytosolic RIG-1 and MDA5, reacting to intracellular parasite motifs [15],[16]; and ii) receptors recognizing cytokines, chemokines, and cell surface proteins produced by the host, e.g., the TNF superfamily. Maturation also leads to the expression of different surface molecules; one of the best markers for human DC maturation is CD83, which is highly expressed by mature DCs both in a membrane-bound and in a soluble secreted form. At least under some circumstances, soluble CD83 seems to have an immunosuppressive effect, as demonstrated by its ability to prevent autoimmune encephalomyelitis in mice [17].

When susceptible (permissive) target cells are not present at the site of viral entry in hosts, cDCs are amongst the first cells encountered by most viruses, simply due to their availability at every possible entry site of the body. This has been formally shown for HIV-1 in an ex vivo organ culture system reproducing the human vagina; in this setting, CD4^+^ T cells present in vaginal epithelium and Langerhans DCs are the first cells invaded by the virus [18]. Viruses that bind DCs activate them to produce pro-inflammatory cytokines and other antiviral molecules and/or get degraded to be presented to specific T cells as peptides. Thanks to this, most viral infections go clinically unnoticed and heal “spontaneously”, being dealt with efficiently by the host's immune system. Certain viruses, however, will successfully replicate in DCs. In this case, these cells will act like other permissive cells with the added consequence that they will migrate across the body, resulting in facilitated spread of infection in addition or as an alternative to activating a defense against it. A third possible outcome of virus–DC interaction is that DCs will not digest and present all the viral particles they have picked up, but will preserve some of them from inactivation so that virions are transported to lymph nodes promoting target cell infection. The overall result of at least certain viral infections can thus be envisioned as a balance between the prevailing activity of DCs in triggering an immune response versus the ability of viruses to exploit DCs or hamper their unique immune functions. This review will mainly focus on the insights brought into the effects viruses exert upon the natural function of DCs during the last few years, while referring to excellent recent reviews for other subjects.

## DC Surface Molecules That Bind Virions

Most viruses exhibit a number of specific features that make them highly immunogenic (Figure 1). DCs have evolved specific surface molecules to uptake pathogens, in addition to expressing a number of specific receptors for certain viruses (such as CD4 and CXCR4 for HIV-1). DC-SIGN (CD209; dendritic cell specific ICAM-3-grabbing non-integrin) and its close relative DC-SIGNR, or L-SIGN found on liver endothelium [19], are some of the most intensively studied pathogen receptors. It is a type II transmembrane C-type lectin specific for mannose and fucose residues that promotes adhesion of DCs to endothelia and is therefore involved in DC trafficking [20]. Although at first considered to be expressed only on cDCs, DC-SIGN was later recognized to be expressed also on B lymphocytes and macrophages, although it has not been detected on the surface of Langerhans cells [19]. DC-SIGN is an ICAM-3 receptor, essential in establishing the initial DC–T cell contact for T cell activation [20]. It was soon understood that DC-SIGN is also a target for pathogens ranging from mycobacteria to major human viruses [20]. DC-SIGN owes this property to the binding of N-linked glycan substituents on viral spikes, thereby enabling virus entry into cell lines expressing low levels of, if any, specific receptors; it may function as a receptor alternative to the specific one or as a non-essential adhesion factor concentrating virions onto cells [21].

Binding of DC-SIGN by several pathogens has also been reported to regulate inflammation and cytokine secretion by DCs via NFκB [22]. In addition, it has recently been shown that DC-SIGN can divert HIV-1 to the proteasome after associating to leukocyte-specific protein 1 in cells infected in vitro, suggesting a role for this molecule in crosspresentation to CD8^+^ T cells [23], while it also has a role in the formation of the infectious synapse (see below). More and more viruses are being found to bind DCs via DC-SIGN, including herpesviruses, Ebola virus, SARS coronavirus, human hepatitis C virus (HCV), and dengue virus (DV) [24]–[29]. It is still unclear whether DC-SIGN and related lectins are actual pathogen-recognition receptors or, rather, antigen-binding receptors whose functions have been subverted by pathogens.

Langerin (CD207) is a membrane marker expressed solely by Langerhans cells, which do not express DC-SIGN [30]. In stark contrast to DC-SIGN, langerin was shown to internalize HIV-1 virions into Birbeck granules and degrade them [31].

Another molecule, DEC-205 (CD205), a 205-kDa membrane molecule that binds certain viruses, was discovered first in the mouse, then in humans. It is a pan-DC-specific surface marker, being expressed at high levels on DCs in the T cell areas of lymph nodes in their steady state and even more when they are mature [32]. In contrast with many endocytosis receptors that only recycle to the cell surface from early endosomes, such as the mannose receptor, targeting antigens to DEC-205 in murine spleen cells allowed them to reach endolysosomes rich in MHC class II products, enhancing the efficiency of antigen presentation to CD4^+^ T cells [32]. Because human DEC205 also proved to be a useful target to prime MHC class I and class II, it is envisioned as a promising candidate molecule for targeting antigens to DCs in vivo with the purpose of priming naïve T cells [33]. However, other surface molecules, like heparan sulfate and Fcγ and mannose receptors, also contribute to the uptake of viruses by DCs [34],[35], and novel virus-binding molecules are discovered continuously on the surface of DCs, the last one being the C-type lectin surface receptor DCIR [36]. The fate of virions encountering a DC is partly determined by the receptors they bind. As shown for HIV, binding specific receptors may determine entry and replication, whereas binding DC-SIGN may lead mostly to transmission of virus to T cells and/or degradation via proteasome resulting in MHC class I presentation, while binding langerin may lead to lysosomal degradation and MHC class II loading. Finally, particular circumstances may affect viral binding and efficiency of infection. For example, semen-derived enhancer of virus infection (SEVI) fibers, which are abundant in human semen, capture HIV virions and promote their attachment to target cells [37]. These molecules may allow virions to bypass uptake by DC-SIGN.

## Pathogenetic Consequences of the Interaction between DCs and Viruses

During viral infection, significant damage to the host can be a “side product” of activating the immune system and of skewing the T helper cell response towards Th1, Th2, Treg, and Th17. Because DCs are pivotal in polarization of the Th response, they are definitely key determinants of the pathogenetic outcome of viral infection and also contribute in determining infectious disease severity.

Certain viruses promote their own persistence by favoring the production of Th1-suppressive cytokines and altering cytokine secretion. Although beneficial in recovery from helminth infestation, Th2 responses are known to have detrimental effects by promoting inflammation, allergy, and fibrosis during viral infection. Triggering of DC-SIGN has been proposed to have a central role in both polarizing the Th response towards Th2 and viral persistence [20],[38]. New evidence shows that DC-SIGN binding by HIV on human cDCs causes activation of Rho guanine nucleotide-exchange factor LARG, which, in turn, is essential for the formation of the infectious synapses and downregulation of IL-12p70 secretion [39]. Thus, activation of DC-SIGN may favor the production of a Th2-related cytokine profile during infection. Another viral strategy to achieve a Th2 environment is to induce DCs and other cell types to secrete Th2-inducing cytokines. The best known example is probably respiratory syncytial virus (RSV): although it does induce DC maturation, virulent RSV strains were shown to inhibit IFN-α and IL-12p70 secretion by human DCs [40]. In mice, RSV infection establishes a Th2 environment that leads to airway infiltration [41] and at least some patients present with signs of Th2-mediated lung injury [40]. In addition, while vaccination attempts in the 1960s with inactivated RSV led to exacerbation of disease, it is interesting that very recently vaccination of mice against RSV was successfully obtained by BCG, a potent Th1 inducer and recombinant for the N and M2 proteins of RSV, and that such protection was found to be mediated mostly by a cellular immune response [42]. A Th2 environment seems so important to viruses that some of them go as far as directly encoding proteins that interfere with immunoregulatory cytokines or proteins with immunomodulatory and/or Th2 cytokine-like activity (i.e., human cytomegalovirus IL10 [43]).

**Figure 2:**
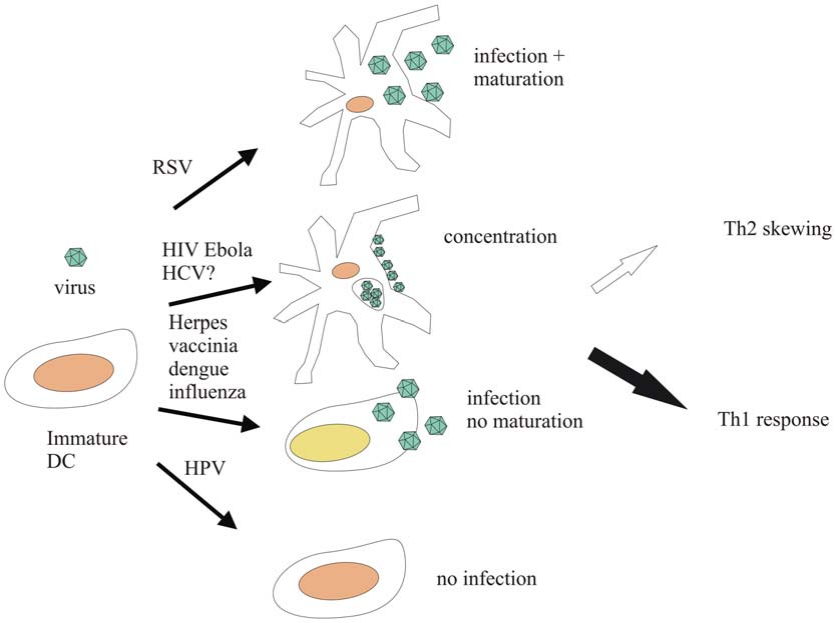
Some effects of viral interaction with DCs. Certain viruses, like RSV, infect DCs, replicate in them, and cause them to mature, while others, like several herpesviruses, dengue virus, and influenza virus, replicate and hinder maturation. Viruses like HPV do not replicate in DCs and are only presented as antigens, while others, like HIV, are concentrated and delivered to their target organs by DCs, which thereby increase the chances of virions to infect cells.

Distinct stages of DC generation may be altered by viral infection, from hematopoietic progenitor differentiation, to maturation, or even both (Figure 2) [38]. In mice, DC progenitors generated under the influence of IL-10 are able to give rise to a DC subset that induces antigen-specific expansion of functional CD4^+^CD25^+^ Treg cells with strong suppressive functions [44]. The role of Treg cells is normally to downregulate immune and inflammatory responses as part of the healing process, but they may be activated to create an immunosuppressive environment during certain viral infections.

Because maturation is essential for DC functionality, there are very few viruses that do not interfere with it. Experiments performed on human monocyte-derived cDCs show that different viruses downregulate distinct stages and events of the maturation process: HCV protein was shown to downregulate expression of HLA-DR [45]; measles virus and RSV rendered immune synapses between cDCs and T cells unstable [46],[47]; and HSV-1 downregulated expression of several costimulatory molecules by cDCs [48], while influenza virus NS1 protein blocked their maturation [49]. On the other hand, other viruses, such as vaccinia virus, may induce cDC maturation, but interfere with antigen presentation nevertheless [50].

The outcome of viral disease is thought to be partly linked to the numbers of and balance between pDCs and cDCs, on the basis of observations made on patients. In patients infected with HSV, severity of disease was found to be inversely correlated to the number of circulating pDCs and to a blunted IFN-α release response during the early phases of infection; clinical data show that patients with HSV-associated acute retinal necrosis have lower numbers of peripheral blood pDCs and exhibit impaired immune responses [51]. Similar findings were obtained in patients infected with DV [52]. Along the same line, during primary HIV-1 infection, pDCs have been found to be reduced in numbers in peripheral blood and such a reduction was associated to higher plasma viral loads [53]. The importance of pDCs has also been demonstrated in the mouse model by preferentially depleting pDCs, but not cDCs, during RSV infection. Again, increased pDC numbers correlated with protection [54]. A reduced number of pDCs may either be genetically determined in given hosts, where viral disease is expected to be consequently more severe due to poor IFN type I response. In other instances, pDC numbers may be virally determined, as suggested by findings showing that patients with chronic hepatitis C exhibited reduced numbers of pDCs as a possible result of apoptosis induced by the interaction of HCV core protein with TLR-2 [55]. Alternatively, a decrease in numbers of pDCs may occur as a consequence of their retention in lymphoid organs, possibly due to higher viral replication in these sites, as suggested for HIV [56]. Interestingly, a recent study suggests that AIDS pathogenesis in humans might stem from failure of adaptive immune responses to control viral replication and from a consequent continuous stimulation of pDCs and NK cells by the substantial amounts of circulating virus [57]. As suggested by the authors, this mechanism might be generalized for viruses reaching high titers in blood and targeting pDCs.

7DC involvement in viral pathogenicity is particularly evident in the lung [54]. In this organ, inflammatory reactions have been well recognized as part of the pathogenic scenario for many viral diseases. It has long been noticed that asthmatic reactions may be linked to pulmonary infection by viruses, especially paramyxoviruses, rhinoviruses, and influenza. Increasing evidence seems to highlight the role of DC-mediated polarization of the immune response in the development of hyperreactivity. When a Th2 cytokine profile is created, IL-4, IL-10, and IL -13 are predominant, and chronic disease is usually evident, whilst recovery from pulmonary infection is generally associated with a Th1 profile [58]. Again, pDCs have been detected at pulmonary sites of viral infection [59]. A recent study describes describes a link between these cells and allergic reactivity in a murine model; DCs were found to upregulate their high-affinity IgE receptor during infection of the lung by Sendai virus, as a result of IFN type I secretion by pDCs. The expression of these receptors is essential to the development of mucous cell metaplasia, one of the signs of airway hyperreactivity [60].

Quite a number of viruses find suitable intracellular conditions to multiply in DCs. The ability to replicate in these cells certainly represents an advantage for them, since DCs are located at the sites of first encounter with the host and may represent the very first site of primary replication. While replicating, viruses may influence DC physiology: DV, for example, gives rise to a complex disease that is strictly dependent on immunopathology. A typical feature of dengue is that greater disease severity is noticed in individuals who have undergone prior infection with a different DV serotype, where antibody-dependent enhancement is believed to be responsible for such a phenomenon, but recent evidence also links it to increased production of DV and pro-inflammatory cytokines by DV-infected mature DCs exposed to DV-immune sera. Such a phenomenon was shown to be mediated by FcγRIIa and downregulated by DC-SIGN expression in human cDCs generated in vitro, suggesting that entry of DV immune complexes via FcγRIIa, but not DC-SIGN, might activate cytokine production [61]. DV is transmitted by direct inoculation of infected saliva from mosquitoes into the host's skin; therefore, skin DCs are probably amongst the first permissive cells encountered expressing high levels of DC-SIGN. In the light of evidence showing that DC precursors capture DV mostly by DC-SIGN and are permissive to virus replication [29],[62], DV infection and replication probably initiates via DC-SIGN, then compounds when antibodies are produced in a secondary heterologous infection as FcγRIIa uptakes DV-antibody complexes.

Herpesviruses are also capable of replication in DCs [63]. They encode a number of early gene products that immediately affect DC physiology to favor immune evasion. Several members of the herpesvirus family attack the expression of costimulatory molecules on the surface of DCs in vitro: HSV-1, for example, induces specific CD83 degradation as early as 6 hours after infection of human cDCs generated in vitro [64]. HSV-1 is known to replicate in DCs, resulting mostly in downregulation of their activity as antigen-presenting cells [63]. Immune dysfunction induced by HSV includes dowregulation of the expression of several costimulatory molecules by mature DCs, mostly mediated by viral proteins directly, together with inhibition of the secretion of soluble Th1 mediators such as IL-12 [63],[64]. Class II-restricted presentation is also hampered by HSV-1 [64]. Similarly, mouse cytomegalovirus alters the balance between stimulatory molecules and inhibitory ones on the surface of infected murine DCs [65]. These immunosuppressive mechanisms may prevail during certain stages of host life and lead to reactivation of herpes infection. However, one must bear in mind that only a percentage of DCs will be infected, while a large fraction of DCs in vivo are expected to perform efficient crosspresentation and priming, as shown for ex vivo DCs from patients infected with cytomegalovirus [66]. HIV also has been reported to preferentially infect a small fraction of blood cDCs [67]. Infected DCs are either prevented from maturing, or HIV-1 may select for a subset of DCs that is resistant to maturation; in any case, HIV-infected DCs exhibit very weak immunostimulatory activity.

## Some Viruses Exploit DCs to Reach Their Target Cells

**Figure 3:**
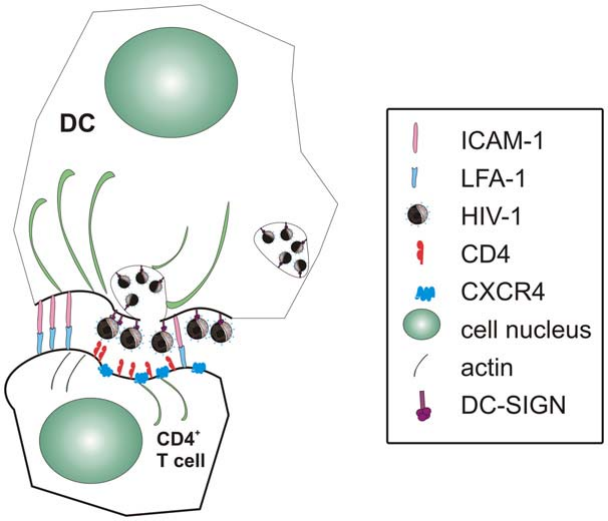
The infectious synapse model. At the site of mucosal entry, DCs concentrate HIV-1 via DC-SIGN. Virions are stored either in “virosomes”, where their infectivity is even preserved, or (and?) on the DC surface and, once in the lymph node, DCs deliver them actively to target CD4^+^ T cells. Several molecules are involved in this event that closely resembles the transmission of acetylcholine along the nerve (synapse), in that virions are actively transported in vesicles along actin fibers and released outside the cells where they immediately find their receptors, which have been concentrated on the T cell side. This mechanism may be active also for other viruses, like HCV and SARS coronavirus.

Lentiviruses, and maybe other viruses as well, have developed an exquisite way of exploiting DC trafficking and functions: they get concentrated into and/or onto DCs at mucosal sites, where they typically make contact with their hosts, then are shuttled to lymph nodes where they get efficiently transferred to T cells of the CD4^+^ phenotype, infecting them. Again, most of our knowledge of lentiviral transfer from DCs to T cells originates from studying HIV-1 in in vitro-generated human cDCs. HIV sequestration by DC-SIGN helps HIV evade immune responses and spread to susceptible cells by a structure called the “infectious synapse” formed at the DC–T cell contact surface (Figure 3) [68]. Engagement of DC-SIGN on the surface of human cDCs by HIV stimulates Rho-GTPase activity, which was found to be essential for the formation of an actin structure giving origin to the infectious synapse and for HIV replication [39]. Actual transfer of HIV from cDCs to T cells has been reported to depend on gp120 and CD4 [69], but receptor clustering and actin tubule formation have long been evident (Figure 3, [68]). Maturation of cDCs seems to enhance virion transfer activity directed at target cells[70].

DC-mediated viral transmission appears to be conserved across lentiviruses infecting different species: simian immunodeficiency virus (SIV) has been shown to be very efficiently transferred to activated T cells by monocyte-derived immature DCs in vitro [71], as has feline immunodeficiency virus (FIV) [72]. This suggests an important mechanism in lentiviral pathogenesis. However, other viruses have been shown to undergo DC-mediated transmission to lymphocytes. Amongst the most recently studied ones, HTLV-1 can infect DCs, which, in turn, can rapidly transfer virus to autologous primary CD4^+^ T cells [73]. DC-mediated transfer of HTLV-1 involves heparan sulfate proteoglycans and neuropilin-1, resulting in productive infection and transformation of the transfected T cells [74]. Even non-lymphotropic viruses such as HCV may exploit this mechanism: HCV-pseudotyped virions have been shown to be transinfected to cells of a hepatoma line after being captured by DCs and cells expressing L-SIGN [75]. Recent studies show that DC-SIGN and CD150 are both involved in direct DC infection by and subsequent transmission of measles virus, indicating a prominent role for DCs during the initiation and dissemination of yet another virus infection [76].

## Can We Exploit DCs to Improve Immune Responses during Vaccination?

Unravelling the details of DC biology brought about hopes for their possible use as immuno-adjuvants and modulators to enhance immune responses against viral infections [77]. Clinical trials evaluating vaccination of patients and experimental animals with ex vivo-generated cDCs pulsed with tumor antigens have provided proof-of-principle that therapeutic effects may be obtained this way. However, this gave rise to surprisingly few studies addressing the concurrent issue as to whether DCs can be used to boost protective immunity to viruses and to skew the immune response towards the direction of healing. Because the generation and maturation process that DCs follow determines their potential of polarizing the T cell response and its direction, different strategies and methods for producing clinically useful DCs should be experimented.

The use of DCs in the therapy of HIV and SIV infection has been explored; in 2004, results were published obtained from untreated HIV-infected patients vaccinated with autologous monocyte-derived DCs loaded with autologous inactivated HIV [78]. A year following vaccination, all patients exhibited plasma viral load decreases that were significantly correlated with their percentages of HIV-specific CD4^+^ and CD8^+^ T cells. To our knowledge, this was the only trial that reported successful use of DCs in the therapy of a human viral disease. More recently, a clinical trial on 18 patients with HIV was carried out by injecting them with monocyte-derived DCs loaded with peptides from Gag, Pol, and Env proteins. Significant increases in T cell immunity were reported, but viremia was not measured [79]. Similarly, human papillomavirus (HPV) 16/18 full-length E7 antigen-pulsed mature autologous cDC vaccination was shown to generate or boost anti-E7 immune responses in patients with cervical cancer, but the overall beneficial clinical effects have not yet been reported [80]. The protocol to generate the DCs for these studies included growth of adherent autologous peripheral blood monocytes in vitro in the presence of granulocyte macrophage colony stimulating factor (GMCSF) and IL-4, and maturation induction after antigen loading with a cocktail of TNFα, IL-1β, and other cytokines, as recommended for clinical-grade DC production.

Because the cat infected by FIV is accepted as a good non-primate model for the study of AIDS, we investigated the vaccination of naïve cats with autologous cDCs generated in vitro and loaded with whole inactivated FIV. In our case, maturation could be achieved by the use of LPS, since feline cytokines were unavailable at the time of the study and this was the only agent able to induce the ability to prime alloreactivity of cat allogeneic T cells [81]. Our results were encouraging, in that all cats exhibited antibody and cellular immunity against FIV antigens, but no protection against challenge with infectious FIV was achieved [82]. In the hypothesis that eliciting memory responses from infected cats might be less demanding than inducing primary responses, we successively tested the same vaccination protocol on FIV-infected cats. In this case, we also assessed viremia levels before and after vaccination, a sustained reduction of viral load being the main goal of the latter approach. Again, we detected increased cellular responses to FIV but no effect on viral load was to be seen [83]. One hypothesis to explain the failure of our DC vaccination protocols to alter the course of FIV infection might be that DCs were not the “suitable” type, in terms of molecules expressed. In this case, it might be interesting to test different protocols for DC generation and maturation to explore the effect of different cytokines and maturation stimuli on the generation of “protective” DCs. This hypothesis is also suggested by recent data obtained in the mouse model that suggests DCs generated in vitro with GMCSF and IL-4 might not be the most appropriate ones to achieve protective immunity activation [11].

Protocols to generate DCs in vitro are often considered too cumbersome to achieve sufficiently high cell numbers and in the appropriate maturation status. Several groups, therefore, aimed at targeting antigens to DCs in vivo with encouraging results [77]. Many reports show that targeting DCs via different receptors (langerin, FcγR, DEC205, and others) improves the ability of different antigens to induce both CD4^+^ and CD8^+^ T cells [84],[85]. Continued studies are needed to assess whether the skewing of the immune response can be carried out by targeting antigens and other surface DC molecules at the same time, and it remains to be seen whether in vitro-derived DCs are efficient not only at inducing immunity but also at directing the immune response to specific types.

## Conclusions

The “iceberg effect”, whereby most viral infections recover with very few, if any, symptoms while a minority of infections lead to serious illness and death, suggests that the most frequent outcome of infection is immune activation, indirectly proving that this is the prevailing role for DCs. However, viruses may also exploit DCs at several steps of their life cycle, limiting immune responses rather than actually suppressing them. Additionally, it is a well-known advantage for viruses to preserve their hosts rather than impoverishing their possible replication reservoir. Thus, a successful virus may be viewed as one that permits immune reactions to take place once a new generation of virions has been released into the host environment. A key event in this respect is the relationship established between viruses and DCs; some viruses can replicate in DCs or merely alter/hijack their functions to their benefit. Shedding light on this relationship might provide means to develop alternatives to classical vaccination strategies in the design of immunotherapeutic approaches to chronic viral infections with global impact such as HCV and HIV. More insight into the field should be gained by in vivo studies aimed at confirming the great deal of results obtained in vitro in the last decade.
